# Influence of enamel and dentin thickness on SpO_2_ readings by pulse oximeter in bleached teeth

**DOI:** 10.1590/0103-644020256818

**Published:** 2026-01-19

**Authors:** Caroline Felippe Fernandes de Souza Nicaloski, Dilma Helena Neves Rodrigues, Julia Menezes Savaris, Elane Lima da Silva, Maria Eduarda Paz Dotto, Cláudia Ângela Maziero Volpato, Lucas da Fonseca Roberti Garcia, Cleonice da Silveira Teixeira

**Affiliations:** 1 Department of Dentistry, Federal University of Santa Catarina, Florianópolis, Santa Catarina, Brazil

**Keywords:** pulse oximeter, dental tissue thickness, tooth bleaching

## Abstract

The objective of this study was to analyze the influence of different enamel and dentin thicknesses on oxygen saturation (SpO₂) readings after dental bleaching. A total of 26 extracted human anterior teeth, sound and without defects, were used. The crown thickness was measured at the middle third using a thickness gauge. Color was evaluated with a spectrophotometer, and SpO₂ readings were obtained through a pulse oximeter coupled to an optical digital simulator, under two perfusion conditions: high (98% SpO₂, 75 bpm) and low (86% SpO₂, 75 bpm). The first reading was performed without the interposition of the dental element, serving as a positive control (L0). After recording SpO₂ and color readings through sound crowns (L1 and C1), the palatal or lingual surfaces were reduced to a thickness of 5 mm (L2), and a new color measurement was performed (C2). The samples were then subjected to dental bleaching, followed by additional color and SpO₂ readings (L3 and C3). Subsequently, the thickness was further reduced to 3 mm, and the measurements were repeated (L4 and C4). Color was evaluated descriptively. Significant differences (p < 0.001) were found between sound teeth (L1) and bleached teeth with 5 mm (L3) and 3 mm (L4) thickness, as well as between unbleached teeth with 5 mm (L2) and the bleached samples (L3 and L4). The findings of this study demonstrated higher SpO₂ readings by the pulse oximeter in bleached teeth. Bleaching had a greater impact on SpO₂ readings than dental thickness, regardless of perfusion condition.

## Resumo

O objetivo deste estudo foi analisar a influência de diferentes espessuras de esmalte e dentina nas leituras de saturação de oxigênio (SpO₂) após clareamento dental. Foram utilizados 26 dentes anteriores humanos extraídos, íntegros e sem defeitos. A espessura das coroas foi medida no terço médio usando um medidor de espessura. A cor foi avaliada com espectrofotômetro e as leituras de SpO₂ foram obtidas através de oxímetro de pulso acoplado a um simulador óptico digital, sob duas condições de perfusão: alta (98% SpO₂, 75 bpm) e baixa (86% SpO₂, 75 bpm). A primeira leitura foi realizada sem a interposição do elemento dental, servindo como controle positivo (L0). Após registrar as leituras de SpO₂ e cor através das coroas íntegras (L1 e C1), as superfícies palatinas ou linguais foram reduzidas a uma espessura de 5 mm (L2), e uma nova medição de cor foi realizada (C2). As amostras foram então submetidas ao clareamento dental, seguido de leituras adicionais de cor e SpO₂ (L3 e C3). Posteriormente, a espessura foi ainda mais reduzida para 3 mm, e as medições foram repetidas (L4 e C4). A cor foi avaliada descritivamente. Diferenças significativas (p < 0,001) foram encontradas entre dentes íntegros (L1) e dentes clareados com 5 mm (L3) e 3 mm (L4) de espessura, bem como entre dentes não clareados com 5 mm (L2) e as amostras clareadas (L3 e L4). Os achados deste estudo demonstraram leituras mais altas de SpO₂ pelo oxímetro de pulso em dentes clareados. O clareamento teve um impacto maior nas leituras de SpO₂ do que a espessura dental, independentemente da condição de perfusão.

**Figure:**
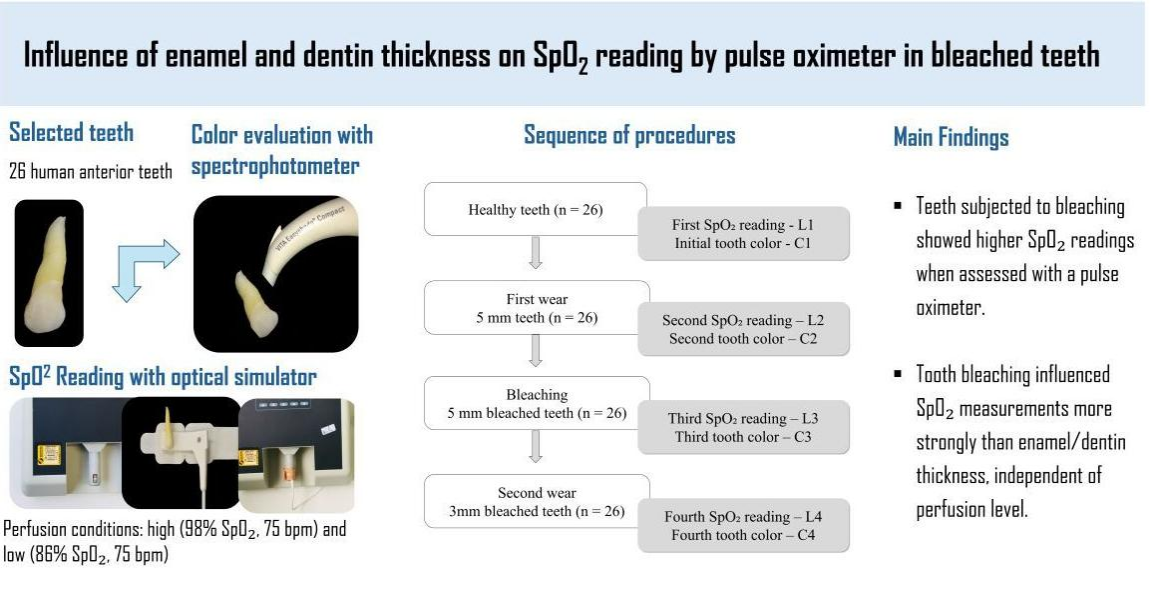

## Introduction

Assessing the physiological state of the dental pulp is essential for endodontic diagnosis^1^. Ideally, the methods employed should be objective, painless, non-invasive, reliable, reproducible, standardized, accessible, and easy to perform^2^. However, the main challenge lies in the physical confinement of the pulp by hard dental tissues, which prevents direct inspection, thereby requiring the use of indirect methods to evaluate pulp vitality^3^. These include thermal, electrical, cavity, and anesthetic tests, as well as clinical and complementary examinations such as radiographs, mobility tests, percussion, palpation, transillumination, and assessment of crown discoloration^2^.

Current methods, such as thermal and electric sensitivity tests, are limited in that they only evaluate neural responses without providing information on pulp blood flow, which plays a vital role in inflammatory processes and tissue repair^4^. Moreover, these tests do not quantify the extent of pulpal disease but merely indicate the presence or absence of a response to external stimuli, and often yield inaccurate results due to their reliance on subjective patient responses, which can be influenced by patient fear and anxiety^5^. Given these limitations and the critical role of blood flow in pulp health, vascular assessments have become increasingly important compared to neural-based evaluations^6^. Several techniques have been explored to assess pulp circulation, including pulse oximetry^7^.

Pulse oximetry (PO) is an objective, non-invasive method based on spectrophotometry and photoplethysmography, commonly used in medicine to measure oxygen saturation and pulse rate through red and infrared light absorption by hemoglobin ^8^ ^,^ ^9^ ^,^ ^10^. Its ability to detect pulsatile blood flow without interference from skin, bone, or venous blood suggests potential for assessing pulp vitality^9^. The effectiveness of a dental-adapted pulse oximeter for this purpose was demonstrated earlier^8^ ^,^ ^9^. However, factors such as dentin thickness, age-related secondary dentin deposition, and light scattering through enamel and gingiva can reduce measurement accuracy^11^ ^,^ ^12^ ^,^ ^13^.

PO is also an effective tool for monitoring pulp health during in-office tooth whitening procedures^14^. These treatments typically use high concentrations (35-38%) of hydrogen or carbamide peroxide to treat dental discolorations^15^. The whitening agents break unsaturated carbon bonds in pigment molecules, enhancing light reflection and reducing color absorption^16^. Due to their low molecular weight and the permeability of enamel and dentin, these agents can diffuse into the pulp chamber, potentially triggering inflammatory responses and tooth sensitivity, and intensifying cytotoxic effects on pulp cells, especially in teeth with thinner enamel^16^ ^,^ ^17^ ^,^ ^18^. Additionally, bleaching has been linked to mineral loss, increased enamel roughness, morphological alterations, and reduced microhardness^19^.

Since it has been observed that the thickness of the tooth structure and bleaching can influence and mask the pulp SpO₂ reading by PO, this study aimed to evaluate the influence of different enamel and dentin thicknesses on the SpO₂ reading performed by the pulse oximeter.

## Materials and methods

This study was carried out after approval of the project number. [blinded for review] by the Local Human Research Ethics Committee. The sample size was estimated based on previously published studies that evaluated SpO_2_ ^4^. For an analysis with α = 0.05 and 80% power, 26 teeth were selected for the experiment.

### Teeth selection and specimen preparation

Twenty-six human anterior teeth, extracted for reasons unrelated to this research, were used. Only healthy permanent teeth with a single root canal were selected. The inclusion criteria included the absence of calcifications, restorations, or visible alterations in enamel and dentin structure, as confirmed by digital radiographs (MicroImagem, Acteon Brasil, Indaiatuba, São Paulo, Brazil) taken in both buccolingual and mesiodistal angulations, along with meticulous inspection under a stereomicroscope (Carl Zeiss AG, Oberkochen, Baden-Württemberg, Germany).

The crown thickness of each tooth was initially measured in the buccolingual direction at a central point in the middle third of the crown, using a thickness gauge (Wilcos, Brasil Indústria e Comércio Ltda., Petrópolis, RJ, Brazil). At this exact point, the initial tooth color (C1) was recorded using an intraoral spectrophotometer (VITA Easyshade®; VITA Zahnfabrik, Bad Säckingen, Germany). For each measurement, the teeth were first dried with absorbent paper and positioned against a standardized white background. The spectrophotometer was programmed to perform three consecutive readings. To ensure the measurement targeted only the central buccal area of each tooth, a heavy-body silicone matrix (3M ESPE Dental Products, St. Paul, MN, USA) was adapted to the probe tip, effectively isolating the region and preventing adjacent areas from influencing the readings. All measurements were conducted by the same operator.

### Reading SpO_2_ through dental crowns

An optical finger simulator for oximetry (Index 2 XLFE, Fluke Biomedical, Everett, Washington, USA) was used to measure SpO₂ through dental crowns. Prior to the experiment, the simulator was calibrated (certificate no. I1573/17). The pulse oximeter employed in the study was the BCI model (Smiths Medical PM Inc., Waukesha, WI, USA), which had also been previously validated for accuracy by the IEB Laboratory at UFSC. SpO₂ readings were taken by a single operator using the PO, with the optical simulator programmed in two distinct modes: high perfusion (HP) and low perfusion (LP). Initially, the simulator was set to HP mode, configured with parameters of 98% SpO₂ and 75 beats per minute (bpm). Subsequently, the LP mode was activated, with parameters set at 86% SpO₂ and 75 bpm. The sensor used for adaptation to the optical finger was the 3025 model (Smiths Medical), consistent with previous studies^8^ ^,^ ^12^ ^,^ ^19^.

The first reading, taken without the interposition of any dental element, served as the positive control (L0). In this step, the 3025 sensor was fully wrapped around the optical finger, with the red light-emitting diode (LED) positioned at the bottom and the receiving diode at the top. Next, each healthy dental crown was positioned with its buccal surface facing the light-emitting diode of the 3025 sensor. This assembly - the sensor and the tooth - was then placed around the optical finger, enabling SpO₂ readings through the crown (L1).

### Reading SpO_2_ through dental crowns after wear

**Figure 1:**
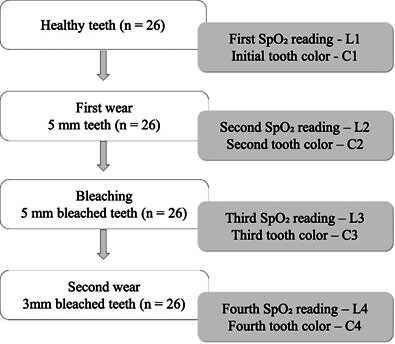
Flowchart illustrating the sequence of procedures and the experimental groups formed at each stage based on the SpO₂ and color readings.

The palatal or lingual surfaces of the teeth were reduced using a conical diamond burr (4138, KG Sorensen, SP, Brazil) operated at high speed under continuous air/water spray. This procedure continued until a thickness of 5 mm was achieved at the central point of the middle third of the crown. At this stage, new color and SpO₂ measurements were taken and recorded as C2 and L2, respectively. Subsequently, all teeth were subjected to a 24-hour whitening procedure using a 16% hydrogen peroxide-based product (Whiteness 16%, FGM, Joinville, SC, Brazil). After the bleaching process, the teeth were rinsed thoroughly under running water. New color and SpO₂ readings were then obtained (C3 and L3). Following this, the crowns were further reduced to a thickness of 3 mm, and a final round of color and SpO₂ assessments was conducted (C4 and L4). A flowchart illustrating the sequence of procedures and the experimental groups formed at each stage based on the SpO₂ and color readings is presented in Figure 1. All SpO₂ values recorded by the pulse oximeter were documented within a maximum interval of 30 seconds (time required for the readings to stabilize). Throughout the experiment, every effort was made to minimize external interferences and ambient noise during the measurements.

### Statistical analysis

The color assessment data were evaluated in a descriptive manner, with colors corresponding to the VITA scale. For evaluating the SpO_2_ readings, after confirming that the data were not normally distributed using the Shapiro-Wilk test (p < 0.05), the Friedman nonparametric test was used to assess whether there were any significant differences between groups. To identify the moments when the SpO_2_ readings varied significantly, a multiple comparison of ordered means was carried out using Dunn's test. The significance level was set at 5%, and SPSS Statistics software (v. 24, SPSS Inc., Chicago, IL) was used for the statistical analysis.

## Results

**Figure 2:**
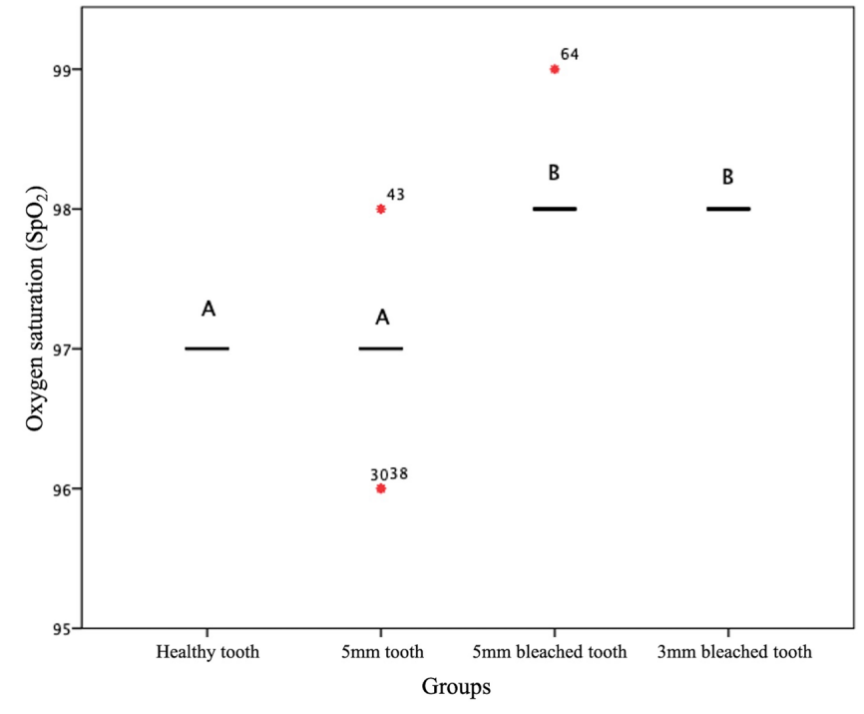
Evaluation of oxygen saturation (SpO_2_) readings according to dentin thickness and bleaching treatment under high perfusion. Distributions identified with capital letters are significantly different at α = 0.05 according to multiple mean comparisons by rank (Friedman test).

**Figure 3:**
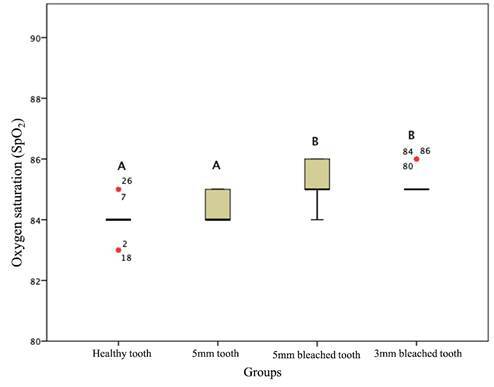
Evaluation of oxygen saturation (SpO_2_) readings according to dentin thickness and bleaching treatment under low perfusion. Distributions identified with capital letters are significantly different at α = 0.05 according to multiple mean comparisons by rank (Friedman test).

The SpO_2_ reading by the PO underwent statistically significant changes (p < 0.05) between the evaluated time points (before and after bleaching) and in the two situations (high and low perfusion). The SpO_2_ reading was statistically different after tooth whitening and a decrease in the thickness of the tooth substrate (Figures 2 and 3). The statistically significant differences, for both high and low perfusion situations, occurred between the groups of healthy teeth (L1) and 5 mm bleached teeth (L3) (p < 0.001); healthy teeth (L1) and 3 mm bleached teeth (L4) (p < 0.001); 5 mm teeth (L2) and 5 mm bleached teeth (L3); and 5 mm teeth (L2) and 3 mm bleached teeth (L4) (p < 0.001). After bleaching, the SpO_2_ values increased significantly (p < 0.05).

As for the analysis of the color of the tooth structures, there were changes in the percentages of each color seen at the different points of evaluation (Table 1). There was a higher percentage of color A1 in healthy teeth (42%) and teeth with 5 mm (34.61%). After bleaching, there was an absence of A4 and C4 percentages and an increase in B1 percentages (34.61% in teeth with 5 mm and 50% in teeth with 3 mm). However, there was no correlation between the color measured and the SpO_2_ values read (Kendall's tau-b, p > 0.05).

**Table 1:** Tooth color percentage (%) across all experimental phases.*

Tooth color	**Healthy tooth (C1)	5mm tooth (C2)	5mm bleached tooth (C3)	3mm bleached tooth (C4)
A1	42.30	34.61	23.07	23.07
A2	15.38	15.38	7.69	15.38
A3	3.84	3.84	11.53	0
A4	3.84	3.84	0	0
B1	26.92	15.38	34.61	50
B2	3.84	19.23	23.07	3.84
B3	3.84	3.84	0	3.84
C1	0	0	0	3.84
C4	0	3.84	0	0

*Percentage of color for each group as they were worn and bleached. C1: color of sound tooth crowns; C2: color of crowns worn down to 5 mm; C3: color of 5 mm crowns after bleaching; C4: color of crowns after bleaching and further reduction to 3 mm.

**The average thickness of sound teeth was 5.39 mm.

## Discussion

The results of the present study demonstrated that bleached teeth showed higher SpO₂ readings when measured with a pulse oximeter. Tooth whitening appeared to exert a greater influence on SpO₂ readings than enamel/dentin thickness, under both low and high perfusion conditions. To standardize the measurements, an optical finger simulator (Index 2 XLFE, Fluke) was used, programmed to simulate SpO₂ levels of 98% (high perfusion) and 86% (low perfusion), with a constant heart rate of 75 bpm. This device can simulate oxygen saturation levels ranging from 35% to 100%^13^ and is widely used for calibrating and verifying oximetry systems^20^. The selected SpO₂ values reflect clinically relevant parameters and align with known limitations of pulse oximetry. Values below 90% are already considered indicative of hypoxemia in clinical settings^21^. Moreover, pulse oximeters are known to have reduced accuracy at perfusion levels below 80%, which justifies the selection of 86% for LP and 98% for HP in this study^8^ ^,^ ^13^.

To reduce the possibility of measurement errors, such as those caused by patient movement or ambient light interference^22^, rigorous control was maintained throughout the data collection process. All readings were taken by a single operator, in a controlled environment, free from external light sources, motion, or noise. Accurate SpO₂ measurements through dental tissues also required the use of a custom-adapted probe that matched the size, shape, and curvature of each tooth. This ensured proper alignment of the light-emitting and receiving diodes, allowing for consistent light transmission through the dental crown^4^. In this study, the 3025 sensor was positioned to fully envelop both the optical finger and the tooth, ensuring standardization and repeatability of the readings.

Based on the results obtained, it can be stated that different enamel and dentin thicknesses showed higher SpO₂ readings after dental bleaching when compared to the values obtained from sound teeth or from teeth with 5 mm thickness prior to bleaching. Interestingly, tooth bleaching appeared to have a greater influence on SpO₂ readings than the thickness of the tooth structure. When comparing healthy teeth to those worn down to 5 mm, no difference in SpO₂ readings was observed. This suggests that a reduction from an average thickness of 5.39 mm (only ~8% thicker) to 5 mm did not significantly impact light transmission. In a previous study, however, a substantial reduction in enamel and dentin thickness, from 4.0 mm to 2.0 mm, which corresponds to a 50 percent decrease, resulted in higher SpO₂ readings^23^. According to those authors, the decreased optical scattering and light absorption within the dental tissues allowed the pulse oximeter sensor to capture the transmitted light more efficiently^23^.

After bleaching, there was a notable increase in SpO₂ values. According to Fried et al.^24^, dentin and enamel absorb little light, with scattering being a more relevant factor in determining light energy distribution within the tissue. Therefore, reduced pigmentation and decreased thickness likely improved light transmission, leading to higher SpO₂ readings. Still, further reduction in thickness after bleaching did not result in increased readings, indicating that bleaching, not thickness alone, was the major contributing factor^13^.

Tooth color is determined by both intrinsic and extrinsic factors. Intrinsically, it is shaped by how light scatters and is absorbed by enamel and dentin. Enamel is translucent and channels light irregularly, while dentin plays a more dominant role in determining color^25^. Extrinsic color is influenced by acquired stains caused by poor oral hygiene, dietary habits, smoking, and exposure to substances such as chlorhexidine and iron salts^26^.

In this study, 5 mm crowns were bleached to standardize color and minimize interference from stains. The whitening process successfully altered tooth color, likely improving light passage and increasing SpO₂ readings. After initial wear, teeth were treated with 16% carbamide peroxide (Whiteness, FGM) for 24 hours. These agents, suitable for at-home use, penetrate easily through enamel and dentin due to their low molecular weight and the natural permeability of these tissues^27^. The whitening gel breaks unsaturated carbon bonds in pigment molecules, reducing their size and enhancing light reflection and transmission^17^. As a result, post-whitening SpO₂ values increased. Contrastingly, a previous study^28^ observed a decrease in SpO₂ values after in-office bleaching with 35% hydrogen peroxide, potentially due to pulpal oxidative stress. Hydrogen peroxide and its derivatives can reach the pulp chamber, cross cell membranes, and produce free radicals, leading to pulp inflammation and decreased oxygen levels^17^. Other factors, such as tissue acidity, vasoconstriction, hemoglobinopathies, and low perfusion, may also reduce SpO₂ values^18^. However, these factors were eliminated in this study, which used an optical finger simulator instead of live tissue.

A clinical study suggests that children's teeth have higher SpO₂ readings due to greater vascularization, which correlates with tooth color and developmental stage^29^. According to Joiner and Luo^30^, deciduous teeth predominantly show lighter shades (A1: 46%, A2: 25%, B1: 11%). These same shades were frequently observed after whitening in this study, supporting the idea that lighter teeth transmit more light, thus enhancing pulse oximeter readings. Estrela et al.^12^ found lower SpO₂ values in older patients (80.0% in those aged 40-44) compared to younger individuals (89.71% in those aged 20-24), likely due to increased dentin thickness with age. However, as highlighted by Joiner and Luo^30^, tooth color tends to darken and yellow with age, which may impact SpO₂ readings more significantly than dentin thickness alone.

Some limitations should be considered when interpreting the results of this study. To minimize bias and improve the internal validity of the measurements, this study employed a controlled perfusion simulator with constant oxygen saturation and standardized measurement procedures, avoiding the physiological variability inherent to human subjects. However, because the experiment was performed in an *in vitro* environment, the conditions do not fully replicate the complexity of clinical situations, such as pulpal microcirculation and dynamic biological tissue responses^28^. Additionally, although the increase in SpO₂ readings observed in this study was statistically significant, it may not be clinically relevant, as such variations should be interpreted as a consequence of optical alterations induced by bleaching rather than as a fundamental change in tissue oxygenation. Therefore, further studies are still needed to verify the influence of tooth whitening and the thickness of hard dental tissues on teeth in vivo.

## Conclusion

The results of this study showed higher oxygen saturation readings by the pulse oximeter in whitened teeth. The whitening of the tooth structure influenced the SpO_2_ reading by the pulse oximeter more significantly than the thickness, both at low and high perfusion.

## Data Availability

The research data are available upon request.
